# Electromagnetic Exposure Dosimetry Study on Two Free Rats at 1.8 GHz via Numerical Simulation

**DOI:** 10.3389/fpubh.2021.721166

**Published:** 2021-09-30

**Authors:** Xianghui Wang, Chengjie Xia, Lu Lu, Hongxin Qi, Jie Zhang

**Affiliations:** Shanghai Key Laboratory of Magnetic Resonance & Biophysics Lab, School of Physics and Electronic Sciences, East China Normal University, Shanghai, China

**Keywords:** electromagnetics, exposure dose, free rats, relative posture position, numerical simulation

## Abstract

Normally, the impact of electromagnetic exposure on human health is evaluated by animal study. The biological effect caused by electromagnetic exposure on such experimental animals as rats has been proven to be dose-dependent. However, though the dose of radio frequency (RF) electromagnetic exposure described by the specific absorbing rate (SAR) on fixed rats has been relatively well-studied utilizing the numerical simulations, the dosimetry study of exposure on free rat is insufficient, especially in the cases of two or more free rats. Therefore, the present work focuses on the variation of SAR caused by the existence of neighboring free rat in the same cage. Here, infrared thermography was used to record the activity of the two free rats who lived in the same cage that mounted at the far-field region in the microwave darkroom for a duration of 48 h. Then, using image processing techniques, the relative positions and orientations of the two rats are identified, which are defined by three parameters, such as the relative distance (*d*), relative direction angle (α), and relative orientation angle (β). Using the simulation software XFdtd 7.3, the influence of *d*, α, and β on the whole-body average SAR (WB-avgSAR) of the rats exposed to 1.8 GHz electromagnetic wave was calculated and analyzed. Then, the average variation of WB-avgSAR of the two rats compared with that of a single rat within 48 h was calculated. The numerical simulation results showed that the relative posture position described by (*d*, α, and β) of the two rats affects their WB-avgSAR and leads to fluctuations at different positions. However, the variation rate of the 48-h-average WB-avgSAR was only 10.3%, which implied that the over-time average SAR of two or more rats can be roughly described by the WB-avgSAR of a single free rat, except when a real-time precise control of exposure dose is necessary.

## Introduction

Public concerns about the potential effects on human health of non-ionizing electromagnetic radiation in the environment require studies on the bioeffect caused by electromagnetic exposure. Normally, experimental animals, such as mice or rats are used to perform biological studies instead of humans. It has been proven that the bioeffect caused by electromagnetic exposure is dose-dependent (1–4), which implies that monitoring the exposure dose in both experimental animals and humans is important. According to IEEE Std C95.1^TM^-2019, the specific energy absorption rate (SAR) is a controlling criterion in the frequency range from 10 kHz to 6 GHz (5). International Commission on Non-Ionizing Radiation Protection (ICNIRP) guidelines for limiting exposure to electromagnetic fields (EMFs) (100 kHz−300 GHz) also indicated that from a health risk perspective, how much EMF power absorbed by the biological tissues was generally interested. Below about 6 GHz, where EMFs penetrate deep into the tissue, SAR, which is the power absorbed per unit mass (W/kg), is useful to describe the dosimetric quantity (6). Since the frequencies of electromagnetic radiation from our daily-used wireless devices, such as mobile phones, radar, and WIFI signals all lie in this range, the bioeffects caused by 10 kHz ~ 6 GHz microwave attract interest. Thus, the studies on exposure dose characterized by SAR under different exposure conditions were carried out through numerical simulation. Besides humans, the exposure doses of various experimental animals, such as mice, rats, rabbits, and monkeys have been studied. In this study, we take rats into account.

The present dosimetry studies indicated that the SAR value of one rat is influenced by both the irradiation manners (e.g., the frequency and the polarization direction of the wave) and the situation of the rat (e.g., the weight, the posture, and the size of the rat). For example, Mason reported that when the RF microwave incident from the back to the abdomen, and the electric component of the microwave polarized along the long axis of the rat, the whole-body average SAR first increased and then decreased with increasing wave frequency within 300–2,060 MHz, and the resonance absorption appeared at a frequency of 600 MHz (7). This implies that the frequency of the electromagnetic wave is a key factor for SAR value. Chen calculated the SAR value under 12 different kinds of irradiation manners of a 220 g rat, whose body size was 244.8 mm in length and 46.8 mm in width. Their results demonstrated that both the incident direction of the wave and the polarization direction of its electric component affect the SAR value of the rat (8). The permittivity of tissues is another major determinant of the SAR value. According to the measurement data from Gabriel, the permittivity values of different organs are different, which also varies with the weight, age, and size of the rat (9–12). Therefore, the weight, age, and size of the rat are also regarded as the influence factors of the SAR value (13).

Meanwhile, it is worth noting that most of the previous studies on electromagnetic exposure dose are based on fixed rats. However, in most real-life events, rats are in a free state rather than in a fixed state, and different postures, such as straight, curling, sleeping, or drinking should affect the SAR value because the polarization direction of the incident microwave changes with rats postures. Therefore, further research on the exposure dose of free rats is necessary to assess accurate exposure dose. One preliminary research work has been carried out concerning a single free rat, and a rough estimation of SAR value was obtained according to the statistical results of rat postures (14). Yet, rats prefer a social living, and the presence of neighboring rats might also inevitably cause variation of the SAR value (15). Thus, it would be meaningful to further study the exposure dose of multiple free rats within a group. To investigate the influence of multiple rats on their whole-body average SAR (WB-avgSAR), Shi et al. separately calculated the average SAR of one, two, and four rats located at a fixed position in a reverberation room. The result showed that the number of rats has no significant influence on the WB-avgSAR of rats, which was 0.0510, 0.0507, and 0.0506 mW/kg, respectively, with 1 V/m spatial electric field intensity and 6 GHz microwave (16). To investigate the whole-body SAR (WBSAR) of newborn and young rats at 2.45 GHz, Wu and coworkers have taken the habit and behavior of the newborn and young rats into consideration. The calculation results showed that the three typical configurations (rats close to the mother, group without mother, and single mother rat) affect the WBSAR of the young rats, with a maximum reduction of 30.0% (17).

To assess the electromagnetic exposure dose closer to the real situation, more and more interest focused on multiple free rats. Though a lot of works have been reported on either single or multiple rats with fixed posture positions, the work on free rat or rats is still insufficient. The present work aimed to study the exposure dose of two free rats. A real-time tracking technique was developed to record the behavior of the two rats and recognize their relative position. The influence of the relative position of the two rats on their WB-avgSAR and the average SAR variation within 48 h was calculated and discussed.

## Methods

### Identification of the Relative Gesture Position of the Two Free Rats

**Figure 1:**
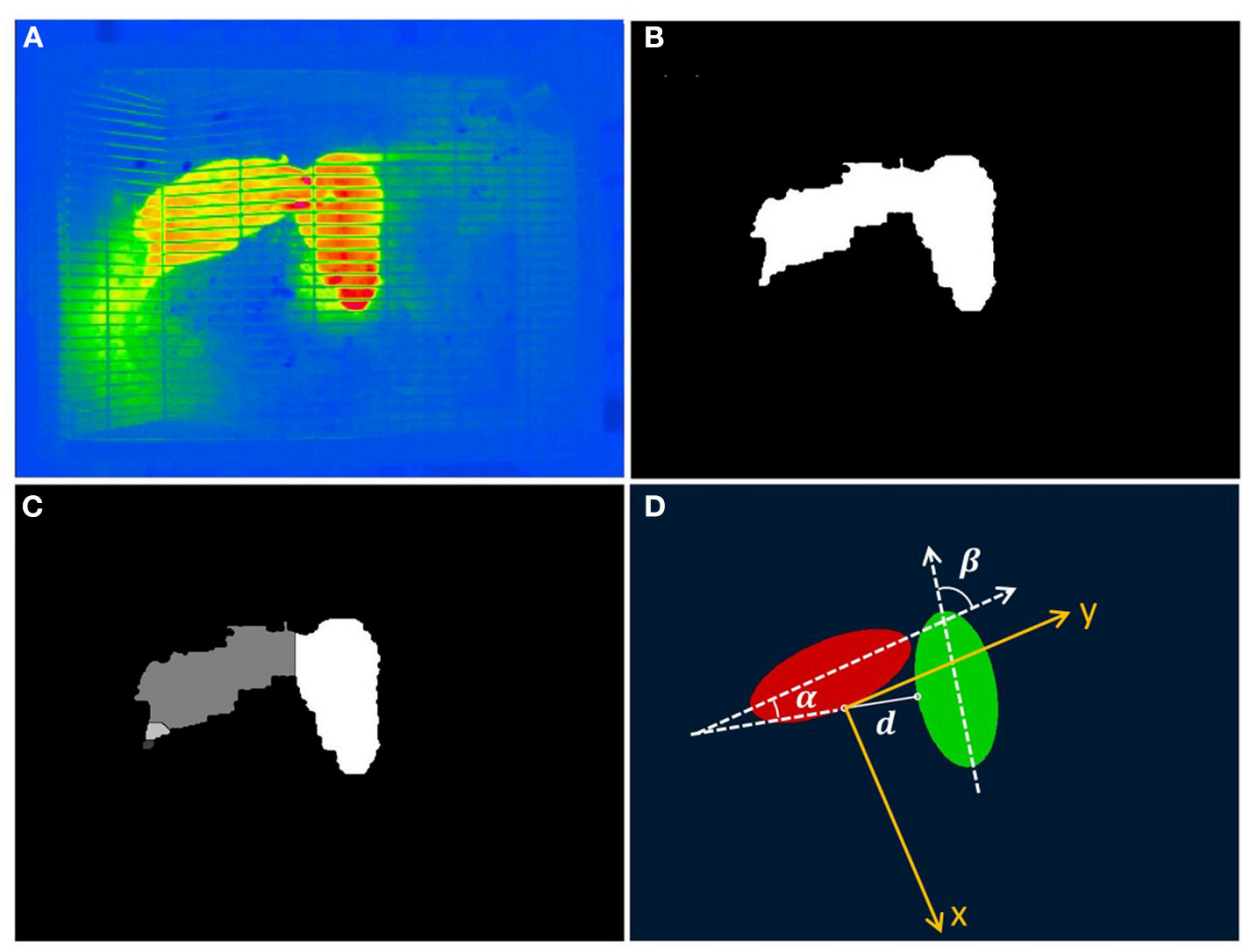
Image processing procedure of identifying images of the two rats. **(A)** A raw thermography. **(B)** The binarized image of **(A)**. **(C)** A segmented image of the two rats with pixels belonging to the different rats shown in different grayscale. **(D)** Representing the positions and orientations of the two rats using two ellipses. The coordinate system and definitions of the relative distances, directions, and orientations are superimposed.

The two rats were feed in a cage (0.46 m × 0.30 m × 0.18 m) with a food and water supply system. The cage was mounted on the sample stage in a microwave darkroom, and the distance between the sample stage and the antenna was 1 m, where the power density was 1.0 ± 0.2 W/m^2^. The microwave was turned on and off every 12 h. An infrared thermography camera (804RC2, Manfrotto Company, Italy) was placed at 1.5 m above the cage to automatically record the behavior of the two rats without disturbing them. Before the 48-h video recording, the rats had adapted to the environment for 3 days. Using an in-house image processing algorithm (18), we distinguished the rough positions and directions of the two rats. The image processing steps were introduced as followed. First, the thermography (Figure 1A) was converted into a gray-scale image by calculating the difference of pixel values of the red channel and the blue channel to obtain a high-contrast image. This gray scale image was then binarized with a properly chosen grayscale threshold (Figure 1B). Next, a watershed method (19, 20) was used to segment and label the images of the two rats, even when they were slightly connected (Figure 1C). Finally, by calculating the secondary moment of the positions of the pixels belonging to each rat, their postures, centroids, and directions can be roughly represented by two ellipses (Figure 1D).

By comparing the results of a series of frames in a row when one of the rats is at rest, we estimate that the uncertainty caused by the above image processing alone is about 3 mm for centroid position and 1.5° for rat direction, respectively. In addition, through visual inspection, we estimate the error caused by different postures of the rats on centroid positions and directions to be <10% of rat length and 10°.

### Characterization of the Relative Gesture Position of the Two Free Rats

The cage was put in the far-field region of the EMF. Therefore, the electric field in the cage area was considered uniform so that the coordinate position of the rat has little influence on its SAR value. Thus, we used three parameters to characterize the relative gesture position of the two free rats. That is, the relative distance d, the relative direction angle α, and the relative orientation angle β.

Through image processing, the two rats were substituted by the two ellipsoid models. Here, we defined the red ellipsoid as rat 1, and the blue one as rat 2. Thus, the two dash lines in Figure 1D show the respective long axes of the two rats. The angle between the two long axes was defined as the relative orientation angle β. Taken the midpoint of the right contour of rat 1 as the coordinate origin O, a plane rectangular coordinates system OXY was established, with its *Y-axis* parallels to the long axis of rat 1. In such a coordinate, the relative distance between the two rats was determined by the length of the connection line between the two midpoints of the two rats, which was marked as *d*. The angle between this connection line and the *X-axis* was defined as the relative direction angle α. With (*d*, α), the relative position of the two rats is determined. By fixing the position of rat 1 at the origin of the OXY coordinate, the posture position of rat 2 relative to rat 1 can be expressed by (*d*, α, β).

### Statistic Analysis of the Relative Gesture Position of the Two Free Rats

Taking rat 1 as the reference, the variation of the relative position caused by the motion of rat 2 was statistically analyzed. Because of the symmetry between the two rats, only the first quadrant of the coordinate system is necessary to represent the relative position of rat 2 referring to rat 1. The quadrant region is partitioned into 16 small regions, with a span of Δ*d* = 0.5λ (λ is the wavelength of the incident microwave, which is 166.7 mm for 1.8 GHz) and Δα = 22.5°. Here, we use *d* = 0 to represent the statistic distance interval of *d*ϵ(0, 0.5λ), and α = 0 to represent the statistic angle interval of αϵ(0, 22.5°). Accordingly, the values of *d* are chosen to be 0, 0.5, 1, 1.5, and 2, respectively, and α were valued as 0, 22.5, 45, 67.5, and 90. Similarly, the relative orientation angle β is categorized into five different numerical ranges marked as β = 0, 22.5, 45, 66.7, and 90° considering the symmetry between rat head and tail which cannot be strictly identified in this work. Therefore, there are 125 combinations of *d*, α, and β in total, and the frequency of each case was calculated based on the 48-h video.

### Numerical Simulation

The rat anatomical model (220 g weight, 244.8 mm long) used here is the same as our previous work (8). XFDTD 7.3 (Remcom, PA, USA) was used to calculate the WB-avgSAR of the rat. The incident power density of the 1.8 GHz microwave was 1 W/m^2^. The boundary condition was set as the perfectly matched layer with a seven-layered absorbance boundary. The grid size was set to 0.4 mm × 0.4 mm × 0.4 mm to guarantee convergent results and accurate SAR values in specific tissues.

**Figure 2:**
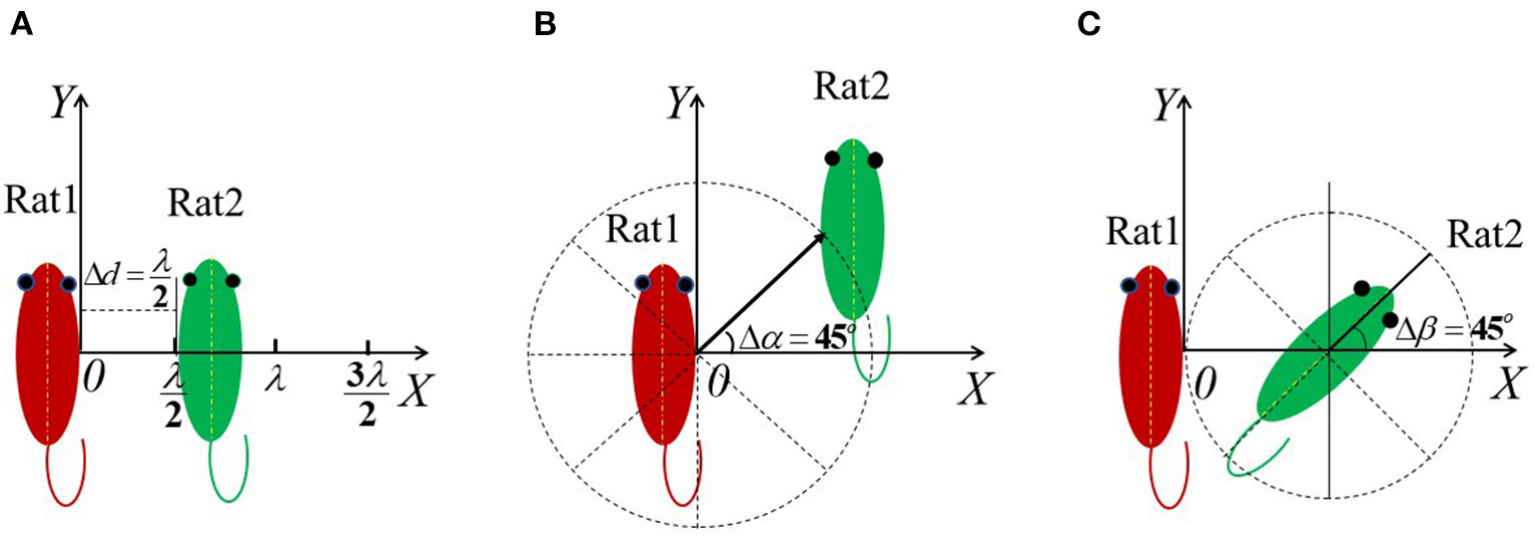
Illustration of the variation manner of *d*, α, and β used for numerical simulation. **(A)** The variation manner of d; **(B)** The variation manner of α; **(C)** The variation manner of β.

To investigate the effect of the relative distance on the WB-avgSAR of the two rats, α and β was set as 0° (in this case, the two long axes are parallel), and the variation span of *d* was set as Δ*d* = 0.5λ. To investigate the effect of relative position on the WB-avgSAR, *d* was set as the half-length of the rat body (i.e., *d* = 122.4 mm in this work) and β = 0°, and α varied from 0 to 360° with a span of Δα = 45°. To investigate the effect of the relative orientation on the WB-avgSAR, the *d* was set as 122.4 mm (to avoid the overlapping of two rats) and α = 0°. Rat 1 located at the coordinate origin and rat 2 laid flatly and spun clockwise with a span of Δβ = 22.5°. The illustration is shown in Figure 2.

Based on the 48-h video, there are 125 combinations of *d*, α, and β in total. Different values of *d*, α, and β were corresponded to the Case ID, as shown in Table 1.

**Table 1:** Case ID corresponding to the 125 relative posture positions of the two rats.

**Case ID**	**Value of *d*, α and β**	**Case ID**	**Value of *d*, α and β**	**Case ID**	**Value of *d*, α and β**
1	*d* = 0, α = 0°, β = 0°	43	*d* = 0.5λ, α = 67.5°, β = 45°	85	*d* = 1.5λ, α = 22.5°, β = 90°
2	*d* = 0, α = 0°, β = 22.5°	44	*d* = 0.5λ, α = 67.5°, β = 67.5°	86	*d* = 1.5λ, α = 45°, β = 0°
3	*d* = 0, α = 0°, β = 45°	45	*d* = 0.5λ, α = 67.5°, β = 90°	87	*d* = 1.5λ, α = 45°, β = 22.5°
4	*d* = 0, α = 0°, β = 67.5°	46	*d* = 0.5λ, α = 90°, β = 0°	88	*d* = 1.5λ, α = 45°, β = 45°
5	*d* = 0, α = 0°, β = 90°	47	*d* = 0.5λ, α = 90°, β = 22.5°	89	*d* = 1.5λ, α = 45°, β = 67.5°
6	*d* = 0, α = 22.5°, β = 0°	48	*d* = 0.5λ, α = 90°, β = 45°	90	*d* = 1.5λ, α = 45°, β = 90°
7	*d* = 0, α = 22.5°, β = 22.5°	49	*d* = 0.5λ, α = 90°, β = 67.5°	91	*d* = 1.5λ, α = 67.5°, β = 0°
8	*d* = 0, α = 22.5°, β = 45°	50	*d* = 0.5λ, α = 90°, β = 90°	92	*d* = 1.5λ, α = 67.5°, β = 22.5°
9	*d* = 0, α = 22.5°, β = 67.5°	51	*d* = λ, α = 0°, β = 0°	93	*d* = 1.5λ, α = 67.5°, β = 45°
10	*d* = 0, α = 22.5°, β = 90°	52	*d* = λ, α = 0°, β = 22.5°	94	*d* = 1.5λ, α = 67.5°, β = 67.5°
11	*d* = 0, α = 45°, β = 0°	53	*d* = λ, α = 0°, β = 45°	95	*d* = 1.5λ, α = 67.5°, β = 90°
12	*d* = 0, α = 45°, β = 22.5°	54	*d* = λ, α = 0°, β = 67.5°	96	*d* = 1.5λ, α = 90°, β = 0°
13	*d* = 0, α = 45°, β = 45°	55	*d* = λ, α = 0°, β = 90°	97	*d* = 1.5λ, α = 90°, β = 22.5°
14	*d* = 0, α = 45°, β = 67.5°	56	*d* = λ, α = 22.5°, β = 0°	98	*d* = 1.5λ, α = 90°, β = 45°
15	*d* = 0, α = 45°, β = 90°	57	*d* = λ, α = 22.5°, β = 22.5°	99	*d* = 1.5λ, α = 90°, β = 67.5°
16	*d* = 0, α = 67.5°, β = 0°	58	*d* = λ, α = 22.5°, β = 45°	100	*d* = 1.5λ, α = 90°, β = 90°
17	*d* = 0, α = 67.5°, β = 22.5°	59	*d* = λ, α = 22.5°, β = 67.5°	101	*d* = 2λ, α = 0°, β = 0°
18	*d* = 0, α = 67.5°, β = 45°	60	*d* = λ, α = 22.5°, β = 90°	102	*d* = 2λ, α = 0°, β = 22.5°
19	*d* = 0, α = 67.5°, β = 67.5°	61	*d* = λ, α = 45°, β = 0°	103	*d* = 2λ, α = 0°, β = 45°
20	*d* = 0, α = 67.5°, β = 90°	62	*d* = λ, α = 45°, β = 22.5°	104	*d* = 2λ, α = 0°, β = 67.5°
21	*d* = 0, α = 90°, β = 0°	63	*d* = λ, α = 45°, β = 45°	105	*d* = 2λ, α = 0°, β = 90°
22	*d* = 0, α = 90°, β = 22.5°	64	*d* = λ, α = 45°, β = 67.5°	106	*d* = 2λ, α = 22.5°, β = 0°
23	*d* = 0, α = 90°, β = 45°	65	*d* = λ, α = 45°, β = 90°	107	*d* = 2λ, α = 22.5°, β = 22.5°
24	*d* = 0, α = 90°, β = 67.5°	66	*d* = λ, α = 67.5°, β = 0°	108	*d* = 2λ, α = 22.5°, β = 45°
25	*d* = 0, α = 90°, β = 90°	67	*d* = λ, α = 67.5°, β = 22.5°	109	*d* = 2λ, α = 22.5°, β = 67.5°
26	*d* = 0.5λ, α = 0°, β = 0°	68	*d* = λ, α = 67.5°, β = 45°	110	*d* = 2λ, α = 22.5°, β = 90°
27	*d* = 0.5λ, α = 0°, β = 22.5°	69	*d* = λ, α = 67.5°, β = 67.5°	111	*d* = 2λ, α = 45°, β = 0°
28	*d* = 0.5λ, α = 0°, β = 45°	70	*d* = λ, α = 67.5°, β = 90°	112	*d* = 2λ, α = 45°, β = 22.5°
29	*d* = 0.5λ, α = 0°, β = 67.5°	71	*d* = λ, α = 90°, β = 0°	113	*d* = 2λ, α = 45°, β = 45°
30	*d* = 0.5λ, α = 0°, β = 90°	72	*d* = λ, α = 90°, β = 22.5°	114	*d* = 2λ, α = 45°, β = 67.5°
31	*d* = 0.5λ, α = 22.5°, β = 0°	73	*d* = λ, α = 90°, β = 45°	115	*d* = 2λ, α = 45°, β = 90°
32	*d* = 0.5λ, α = 22.5°, β = 22.5°	74	*d* = λ, α = 90°, β = 67.5°	116	*d* = 2λ, α = 67.5°, β = 0°
33	*d* = 0.5λ, α = 22.5°, β = 45°	75	*d* = λ, α = 90°, β = 90°	117	*d* = 2λ, α = 67.5°, β = 22.5°
34	*d* = 0.5λ, α = 22.5°, β = 67.5°	76	*d* = 1.5λ, α = 0°, β = 0°	118	*d* = 2λ, α = 67.5°, β = 45°
35	*d* = 0.5λ, α = 22.5°, β = 90°	77	*d* = 1.5λ, α = 0°, β = 22.5°	119	*d* = 2λ, α = 67.5°, β = 67.5°
36	*d* = 0.5λ, α = 45°, β = 0°	78	*d* = 1.5λ, α = 0°, β = 45°	120	*d* = 2λ, α = 67.5°, β = 90°
37	*d* = 0.5λ, α = 45°, β = 22.5°	79	*d* = 1.5λ, α = 0°, β = 67.5°	121	*d* = 2λ, α = 90°, β = 0°
38	*d* = 0.5λ, α = 45°, β = 45°	80	*d* = 1.5λ, α = 0°, β = 90°	122	*d* = 2λ, α = 90°, β = 22.5°
39	*d* = 0.5λ, α = 45°, β = 67.5°	81	*d* = 1.5λ, α = 22.5°, β = 0°	123	*d* = 2λ, α = 90°, β = 45°
40	*d* = 0.5λ, α = 45°, β = 90°	82	*d* = 1.5λ, α = 22.5°, β = 22.5°	124	*d* = 2λ, α = 90°, β = 67.5°
41	*d* = 0.5λ, α = 67.5°, β = 0°	83	*d* = 1.5λ, α = 22.5°, β = 45°	125	*d* = 2λ, α = 90°, β = 90°
42	*d* = 0.5λ, α = 67.5°, β = 22.5°	84	*d* = 1.5λ, α = 22.5°, β = 67.5°	–	–

In the 25 cases of *d* = 0 (α and β varied from 0 to 90°), the two rats tightly closed to each other or even overlapped. In such cases, it is difficult to separate the gesture of the two rats in XFDTD software. Therefore, we took the 25 cases into 1 case consideration that valued as *d* = 0, α = 0, and β = 0 in the numerically simulated. The WB-avgSAR of rat 1 in each case was recorded as *S*_1i_, where *i* varied from 1 to 125. Then, η_*i*_ was calculated with Equation (1)


(1) $$\begin{array}{r} \eta_{i}\text{=}\frac{S_{1i}-S_{\text{1}}}{S_{1}}\times 100\% \end{array}$$

where *S*_1_ is the WB-avgSAR of rat 1 fixed on the coordinate origin with the irradiation manner of EHK, which takes the value of 0.043 W/kg here. η_*i*_ reflects the influence of the relative gesture position of rat 2 on the WB-avgSAR of rat 1.

Two irradiation methods, EHK (the microwave incident from the back to the abdomen of the rat, and its electric field polarization direction was along the long axis of rat 1) and HEK (the microwave incident from the same direction, but its electric field polarization direction was along the short axis of rat 1), were considered in this study.

The average variation of the WB-avgSAR (η_*avg*_) of rat 1 within 48 h was subsequently calculated by Equation (2) to see the overall impact of the existence of rat 2:


(2) $$\begin{array}{r} \eta_{avg}=\underset{i}{\sum }W_{i}\times \eta_{i}\; \end{array}$$

where, *W*_*i*_ is the statistical probability of each relative posture position obtained from the 48-h-video.

## Results

### The Effect of the Relative Position on the WB-AvgSAR of the Two Rats

**Figure 3:**
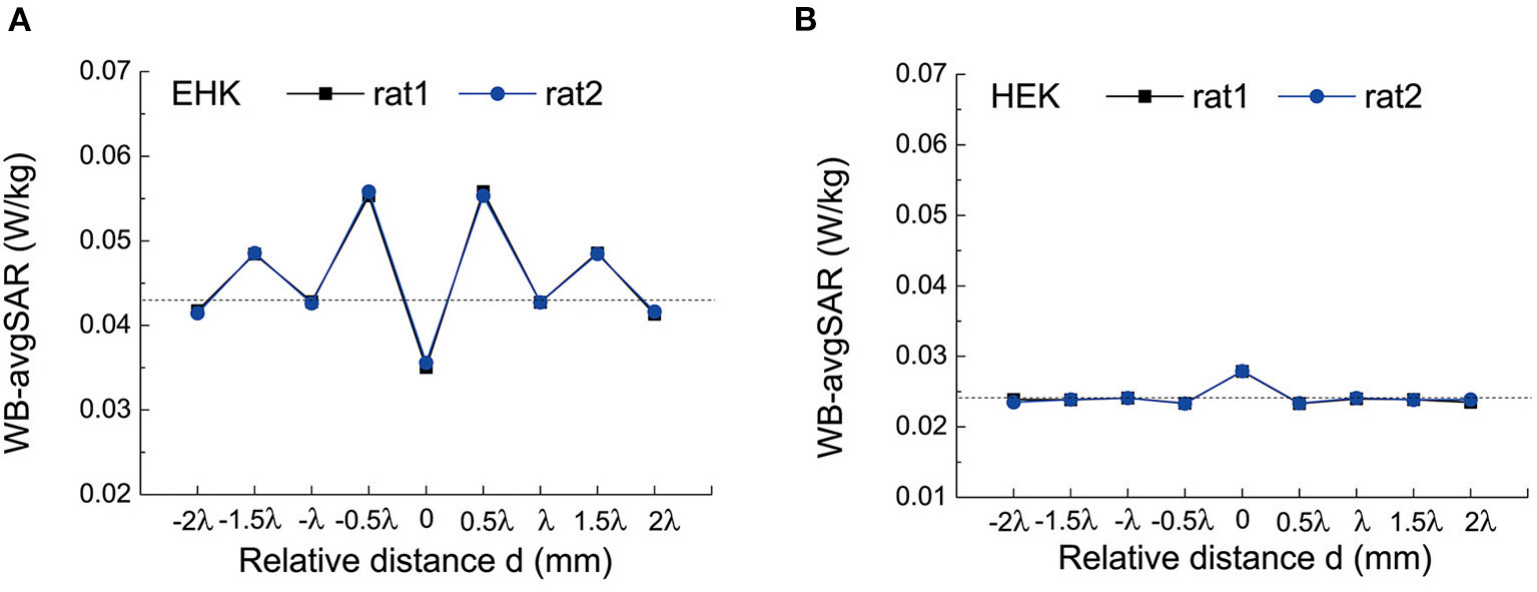
The whole-body average SAR (WB-avgSAR) of two rats in different horizontal distance *d*. **(A)** The simulation results under EHK; **(B)** The simulation results under HEK.

The influence of the relative distance between the two rats on the WB-avgSAR is shown in Figure 3. The dashed line indicates the WB-avgSAR of a single rat, which has been calculated as 0.043 W/kg for EHK and 0.0245 W/kg for HEK. The WB-avgSAR of both rats is the same because they two are plane symmetry. The distance between the two paralleled rats does influence their WB-avgSAR in a form of fluctuation. In both irradiation manners EHK and HEK, the maximum values appeared at $\text{d}=\frac{2k-1}{2}\lambda$, while the minimum values appeared at *d* = kλ. The amplitude of the fluctuation decreased with the relative distance increasing. This might result from the disturbance of the spatial distribution of the microwave due to the existence of the neighboring rat. Compared with HEK, the results of the EHK method showed a higher WB-avgSAR and a larger fluctuation amplitude, this might be since EHK contributes to a long-axis polarization direction of its electric field.

**Figure 4:**
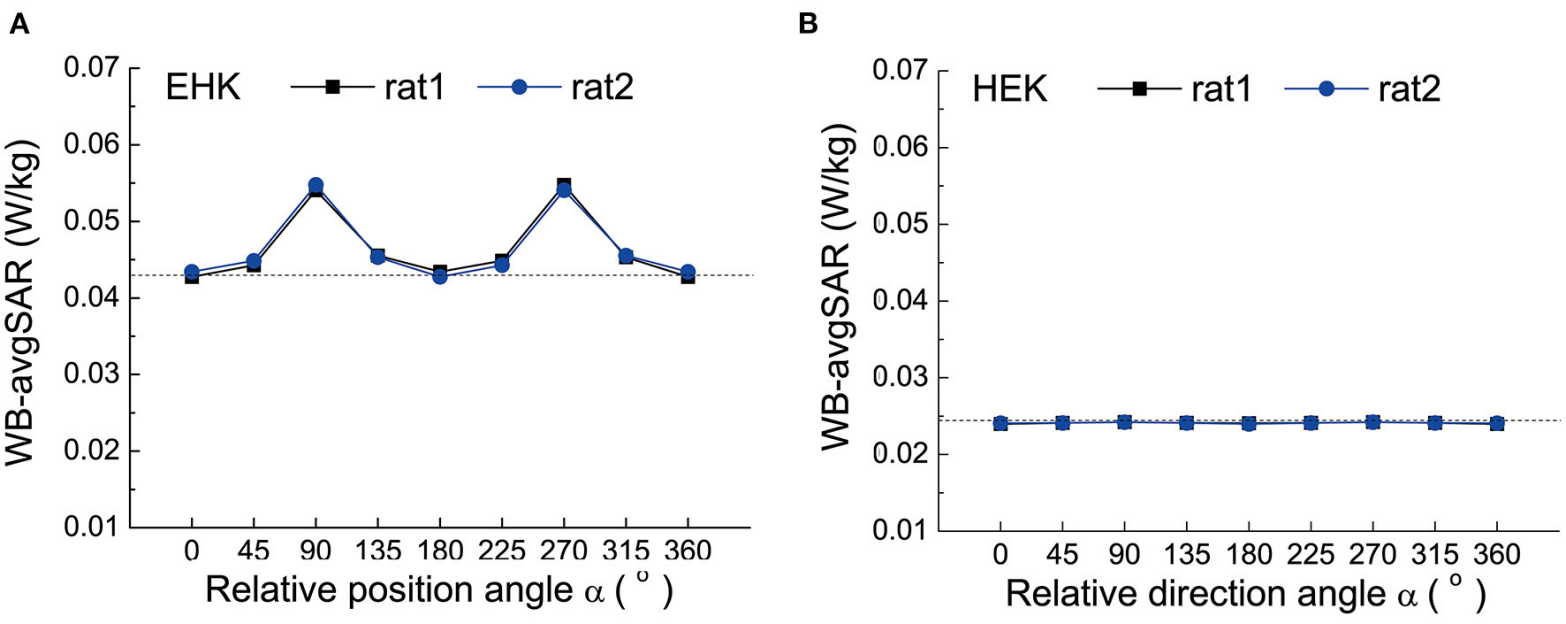
The WB-avgSAR of two rats in different relative direction angles α **(A)** The simulation results under EHK; **(B)** The simulation results under HEK.

The influence of relative direction angle α on the WB-avgSAR is shown in Figure 4. Similar to the effect of d, the WB-avgSAR of EHK irradiation was higher than that of HEK due to electric field polarization direction. The effect of α exhibited an orientation symmetry pattern in the case of EHK, and the maximum value appeared at the two rats in alignment that correspond to α = 90° and 270°.

**Figure 5:**
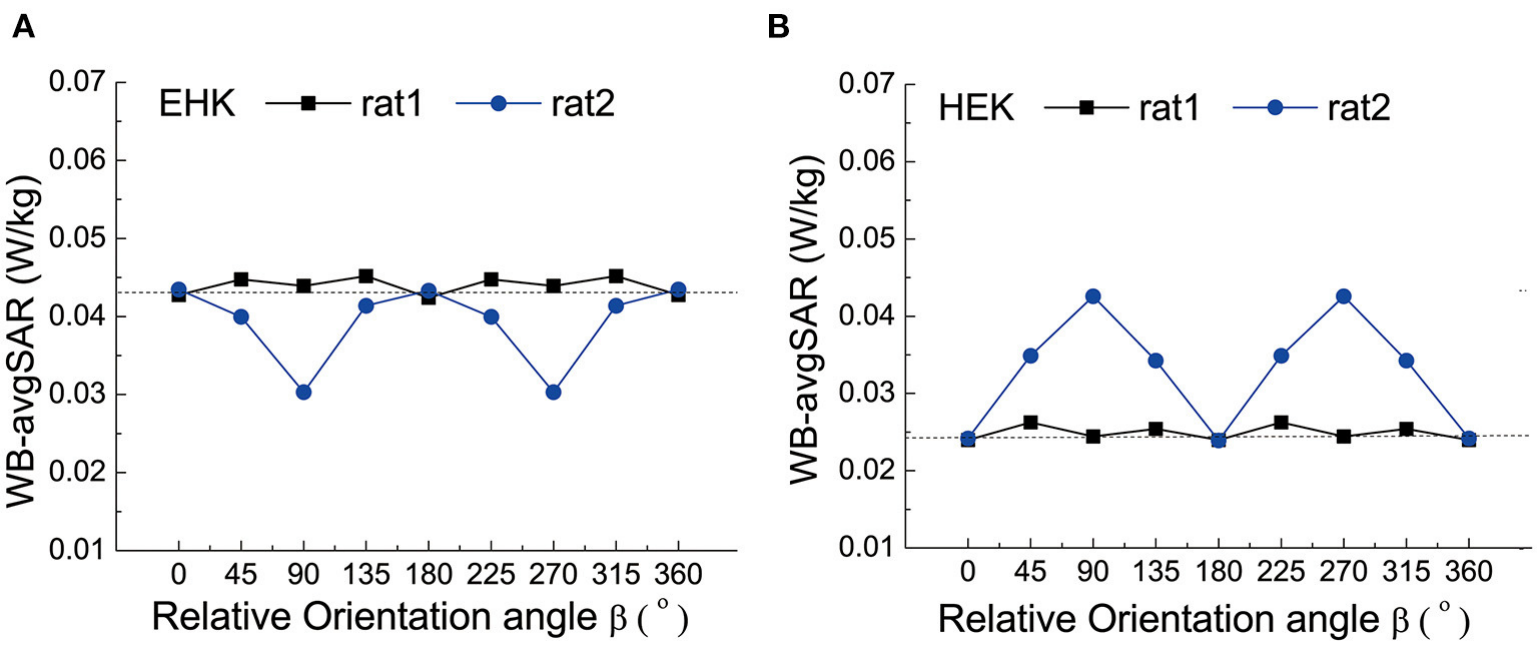
The WB-avgSAR of two rats in different relative orientation angles β. **(A)** the simulation results of rat 1 laid flatly and in EHK radiation mode; **(B)** The simulation results of rat 1 laid flatly and in HEK radiation mode.

The influence of relative orientation angle β on the WB-avgSAR was shown in Figure 5. Since rat 1 was in a fixed position, the relative orientation of rat 2 leads to the asymmetric fluctuation of the WB-avgSAR on rat 1. The WB-avgSAR of rat 2 varied with the orientation angle β because its long-axis changed with β. When its long-axis was aligned with the electric field polarization direction under the β = 90° and 270°, the WB-avgSAR value of rat 2 is maximized.

### The Statistical Result on the Relative Gesture Position of the Two Rats Within 48 h

**Figure 6:**
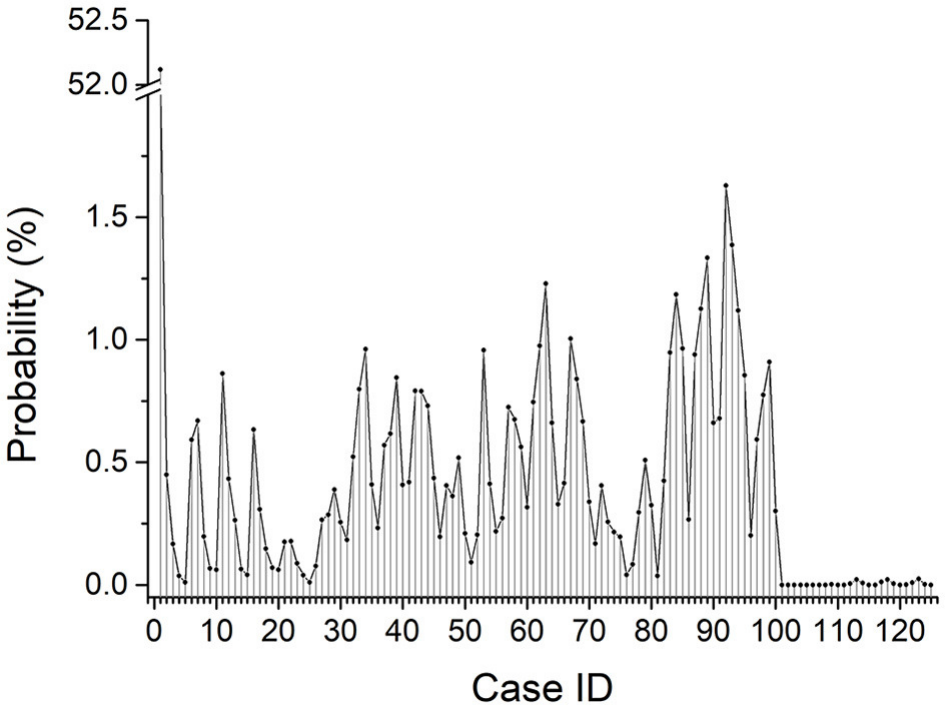
Time probability distribution of two free rats in 125 relative posture positions (total time duration was 48 h).

The statistical result on the relative gesture position of the two rats within 48 h was shown in Figure 6. It gives the value of *W*_*i*_ in Equation (2).

For most of the time, the two rats laid closely, and the total probability of *d* = 0 (corresponded to case 1 to case 25) is about 57.74%. For the rest of the time, the two rats moved freely in the cage, and the distance between them mostly ranged from 0.5λ to 1.5λ. The probability of each relative gesture position fluctuated between 0 and 1.63%.

### The Effect of the Existence of Rat 2 on the WB-AvgSAR of Rat 1

**Figure 7:**
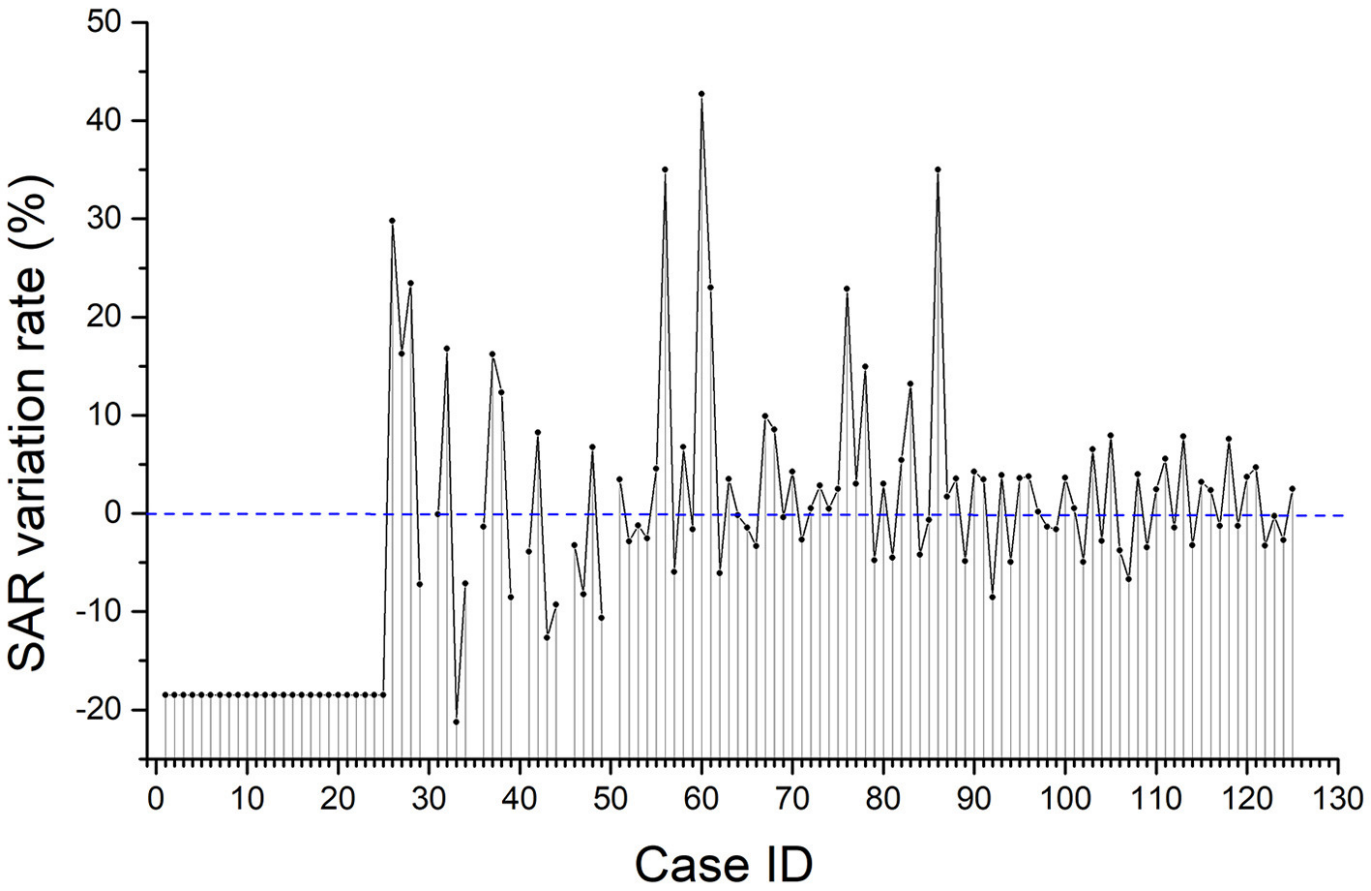
The variation rate of the WB-avgSAR of rat 1 under each relative gesture position.

The variation rate of the WB-avgSAR of rat 1 under each relative gesture position case that was calculated from Equation (1) is shown in Figure 7. The existence of rat 2 affects the WB-avgSAR of rat 1 to a certain degree, and the effect would be either positive or negative. In most cases, the variation rate is lower than 10%. The average variation rate of the WB-avgSAR of rat 1 given by Equation (2) is about −10.3% within 48 h. When there are two free rats in the cage, the WB-avgSAR of each rat reduced about 10.3% when compared with the case of only one rat in the cage. However, it is worth noting that the calculation error caused by theoretical simulation is normally larger than 10%. So that, the variation of the WB-avgSAR of the rat that caused by the existence of another rat is too small to be considered unless the real-time SAR is required to be precisely controlled.

## Discussion

Wu et al. reported that the exposure of multiple rats led to significantly different dosimetric results at the microwave frequency (17), and they further indicated that the variation of the WB-avgSAR of the rat due to the existence of the adjacent rats differed from their intervals (21). Our study on the effect of the relative positive of the two rats showed similar results.

At a certain moment, the relative gesture position of two free rats is definable and can be treated as the two fixed rats. Thus, in the case of two free rats, the real-time dosimetric variation depends on their relative gesture position at that moment. Since the real-time dosimetric variation could be both positive and negative, the time-weighted average dosimetric variation within 48 h was only 10.3%.

Most of the time, the two rats stayed closely or even overlap. In such cases, the two rats might absorb the irradiation power together with a minimum reflection area, which led to a negative effect on the WB-avgSAR. Therefore, though in some cases, the positive effect could exceed 40%, the overall effect of the existence of rat 2 is to reduce the WB-avgSAR of rat 1.

In the present study, we only considered the average dosimetric variation on the WB-avgSAR of the rat. The dosimetric variation within the different organs might be further studied.

## Summary

Infrared thermography was used to record the activity of the two free rats who lived in the same cage that mounted at the far-field region in the microwave darkroom. Using an in-house image processing algorithm, we distinguish the rough positions and directions of the two rats. Three parameters as the relative distance (*d*), relative direction angle (α), and relative orientation angle (β) were defined to describe the relative posture position of the two rats. The existence of rat 2 leads to a fluctuation on the WB-avgSAR accompany by the change of *d*, α, and β. However, the variation rate of the 48-h-average WB-avgSAR is only around 10%, which implies that the over-time average WB-avgSAR of two free rats can be roughly described by the SAR of one free rat unless the real-time precisely control of exposure dose is required.

## Data Availability Statement

The original contributions presented in the study are included in the article/Supplementary Material, further inquiries can be directed to the corresponding author/s.

## Ethics Statement

The animal study was reviewed and approved by the Animal Experimental Ethics Committee of East China Normal University.

## Author Contributions

XW participated in the method establishing to characterize the relative gesture position of the two rats, and the analysis of calculation result and wrote the manuscript. CX designed the image processing algorithm to provide the statistics on rats' relative positions and orientations. LL performed the video recording, performed the calculation by using XFDTD. HQ participated in the program writing, the numerical simulation, and the calculation result reviewing. JZ participated in the study design and reviewed the results. He also participated in the adjustment of devices and methods establishing to characterize the relative gesture position of the two rats. All authors contributed to the article and approved the submitted version.

## Funding

This study was funded by the National Natural Science Foundation of China (31600675).

## Conflict of Interest

The authors declare that the research was conducted in the absence of any commercial or financial relationships that could be construed as a potential conflict of interest.

## Publisher's Note

All claims expressed in this article are solely those of the authors and do not necessarily represent those of their affiliated organizations, or those of the publisher, the editors and the reviewers. Any product that may be evaluated in this article, or claim that may be made by its manufacturer, is not guaranteed or endorsed by the publisher.
